# The Combination of Phage Therapy and β-Lactam Antibiotics for the Effective Treatment of *Enterococcus faecalis* Infections

**DOI:** 10.3390/ijms26010011

**Published:** 2024-12-24

**Authors:** Magdalena Moryl, Paulina Szychowska, Julia Dziąg, Antoni Różalski, Agnieszka Torzewska

**Affiliations:** Department of Biology of Bacteria, Institute of Microbiology, Biotechnology and Immunology, Faculty of Biology and Environmental Protection, University of Lodz, 90-237 Lodz, Poland; szychowska78@gmail.com (P.S.); juladziag@gmail.com (J.D.); antoni.rozalski@biol.uni.lodz.pl (A.R.); agnieszka.torzewska@biol.uni.lodz.pl (A.T.)

**Keywords:** *Enterococcus faecalis*, phage–antibiotic synergy, β-lactam antibiotics, urinary tract infections

## Abstract

A phage–antibiotic synergy could be an alternative in urinary tract infection (UTI) therapy, as it leads to the elimination of bacteria and to the reduction in variants resistant to phages and antibiotics. The aims of the in vitro study were to determine whether phages vB_Efa29212_2e and vB_Efa29212_3e interact synergistically with selected antibiotics in the treatment of *E. faecalis* infections, to optimize antibiotic concentrations and phage titers for the most effective combinations, and to assess their impact on the number of spontaneous resistant variants and on the phages’ reproductive cycles. The modified double-layer disc diffusion method, checkboard, time–kill assays, one-step growth method and the double agar overlay plaque assay were implemented. Synergistic interactions were most often observed after the combined action of phages 2e or 3e and β-lactam antibiotics on *E. faecalis* strains. The beneficial effects depended on the bacterial strain, phage and antibiotic used. The lowest minimum inhibitory concentration (MIC_50_) values of the antibiotics were recorded, after the application of low titers of phage 2e, and high titers of phage 3e. The combined use of the tested agents resulted in a significant reduction in the number of resistant variants and had an impact on the reproductive cycle of the tested phages, e.g., a 50% increase in burst size, and a 5 min reduction in the latency period of 2e were observed. The study confirmed beneficial interactions between phages and β-lactam antibiotics against *E. faecalis* growth.

## 1. Introduction

Enterococci are Gram-positive, facultative anaerobic bacteria which colonize the gastrointestinal tract as a part of natural human intestinal microbiota, but are also known uropathogens [1,2]. They are a leading cause of different healthcare-associated infections, e.g., endocarditis, bacteremia, pneumonia, endocarditis, and urinary tract infections (UTIs) [3,4,5]. They contribute to over 30% of nosocomial UTIs and have been identified as the second leading pathogen in catheter-associated urinary tract infections (CAUTIs) [6]. Enterococcal prevalence in hospitals is related to their multidrug antibiotic resistance as well as tolerance to a broad spectrum of drugs and their ability to survive in extreme, heavily disinfected environments for long periods of time [6]. Therefore, the nosocomial infections caused by enterococci pose a treatment challenge. Enterococci have highly malleable genes, which enable them to easily acquire mobile genetic elements, form hybrid genomes with other enterococci, and transfer genes across species, including diverse bacteria. Furthermore, the acquisition of the genes is facilitated by the absence of genomic defense mechanisms: resistance-modification systems and CRISPR-Cas [7,8]. This genetic flexibility is the primary reason for the rapid evolution of extensive resistance to many antimicrobials (e.g., vancomycin). Enterococci also exhibit natural resistance to various antibiotics, including quinolones, oxazolidinones, and β-lactams (cephalosporins) [6].

Despite rising bacterial resistance to antimicrobials, the treatment of UTIs is mainly based on the use of antibiotics. The preferred options for uncomplicated UTI treatment are cotrimoxazole, fosfomycin and nitrofurantoin, or as second choice, amoxicillin with clavulanic acid, or fluoroquinolones, used as a last resort [9]. In the case of a complicated UTI, intravenous ampicillin, fluoroquinolones, oxazolidinones, vancomycin, or daptomycin can be applied. Combination therapy with antibiotics and β-lactamase inhibitors is also commonly used [6]. A relatively new idea in the treatment of UTIs is synergistic therapy with bacteriophages and antibiotics. It is a researcher’s proposal for the increasing resistance of bacteria [10,11].

The phenomenon of phage–antibiotic synergy (PAS) has many benefits including more effective elimination of bacteria than the separate use of the agents, improved penetration into biofilms, reduction in the appearance of phage- and/or antibiotic-resistant mutants, an increase in the sensitivity of bacteria to antibiotics—a decrease in the minimum inhibitory concentration (MIC) of antibiotics and an increase in the size of plaques, faster amplification of phages and a larger virion release [12,13,14]. Various mechanisms have been proposed to explain that phenomenon. One of the hypotheses postulates that bacteriophages improve antibiotic diffusion and penetration to a bacterial cell. Phage enzymes (polysaccharide depolymerases) play a significant role in this process because they destabilize the bacterial cell wall and enable drug diffusion [14]. Another hypothesis suggests that bacterial lysis mediated by phages leads to a decrease in bacterial population, which reduces the selection pressure for the development of antibiotic resistance. It should also be taken into account that phages and antibiotics generate diverse selection pressures on various bacterial subpopulations, and lead to a synergistic effect because their combined action eliminates a larger number of resistant strains [14,15,16]. A mechanism known as the “evolutionary trade-off” suggests that the selective pressure exerted by phages accelerates bacterial mutations, which help the bacteria to evade phage infection, but at the same time affects their fitness [17]. Phages bind to bacterial cells via receptors, such as lipopolysaccharides, teichoic acids, proteins, flagella or capsules. These receptors are often typical growth or virulence factors or antibiotic resistance-related components. For instance, a membrane protein acting as a part of a drug efflux pump may serve as a phage receptor. During phage infection, bacteria mutate under phage selection pressure and alter the structure of this membrane protein. While this mutation confers bacterial resistance to the phage, it simultaneously inactivates the efflux system, one of the key mechanisms of antibiotic transport from the bacterial cell, which leads to the resensitization of bacteria to the antibiotic [12,17]. PAS could be also the result of antibiotic-induced changes in bacterial physiology which increase the phage activity [18]. Sublethal doses of both β-lactam antibiotics and fluoroquinolones often lead to cell division disorders: elongation, swelling or cell filamentation [18,19]. The mechanism of this phenomenon varies depending on the antibiotic and is most often related to penicillin-binding proteins (PBPs) or the SOS system [20]. Elongation or filamentation of bacterial cells affects the reproductive cycle of phages and leads to a shortened adsorption time and latency period, often combined with an increased burst size [11,12].

The aim of the in vitro study was threefold: (1) to determine whether phages vB_Efa29212_2e and vB_Efa29212_3e interact synergistically with selected antibiotics used in the treatment of urinary tract infections; (2) to optimize antibiotic concentrations and phage titers for the most effective combinations; and (3) to assess the impact of combined therapy on the antibiotic dose reduction required to significantly inhibit the growth of uropathogenic *E. faecalis* strains, on the number of resistant forms generated, and on the biological features of the bacteriophages.

## 2. Results

### 2.1. Bacteriophages Host Range

A total of 33 *E. faecalis* strains were tested to evaluate the host range of the bacteriophages studied. Table 1 presents the results obtained. Phages vB_Efa29212_3e exhibited a lytic activity against 31 of the tested bacterial strains, which indicates a broad host range. However, only in the case of eight *E. faecalis* strains, fully clear zones in the lawn after the phage application at a routine test dilution (RTD) of 10^−2^ or 10^−3^ were observed. Bacteriophage vB_Efa29212_2e replicated only in four *E. faecalis* strains, indicating a narrow host range, with clear zones in the lawn for two of the strains (RTD 10^−3^).

Based on the results obtained, one reference bacterial strains and ten clinical strains in which phages were easily replicated were selected for further studies. 

### 2.2. A Modified Double-Layer Disc Diffusion Method

**Figure 1:**
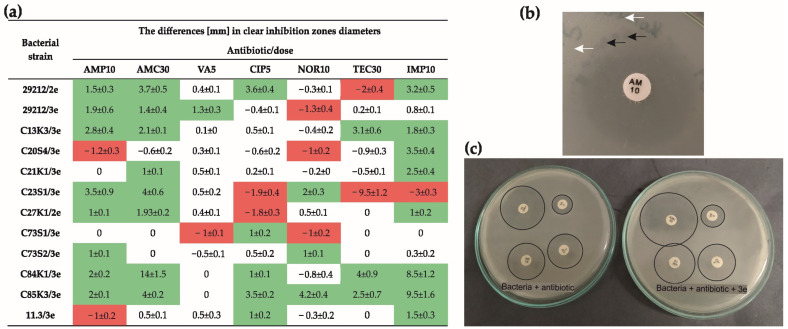
Disk diffusion method in the phage and antibiotic action against *E. faecalis* strains. (**a**) The differences obtained in clear zone diameters (in mm) after the application of antibiotics alone and antibiotics with phages. Green marked fields indicate the possibility of synergy, red marked fields indicate the possibility of antagonism. (**b**) The sample visualization of the morphology of phage plaques in the antibiotic-active zone and in the antibiotic-free zone. The white arrows indicate a typical plaque of the phage strain; the black arrows indicate plaques of increased size. (**c**) Visualization of the occurrence of PAS effect (increased inhibition zones after phage implementation). AMP or AM—ampicillin, AMC—amoxicillin/clavulanic acid, VA—vancomycin, CIP—ciprofloxacin, NOR—norfloxacin, TEC—teicoplanin, IMP—imipenem.

The possibility of the occurrence of phage–antibiotic synergy was assessed by comparing the diameters (in mm) of clear inhibition zones after the application of antibiotics alone and in combination with phages on an *E. faecalis* lawn and the differences in the morphology of phage plaques in the antibiotic-active and antibiotic-free zones (Figure 1). Possible synergistic interactions (evidenced by an increase in inhibition zone size and larger phage plaques) and antagonistic interactions (characterized by a decrease in inhibition zone size and no changes in phage plaque morphology) between the phages and antibiotics were observed. In Figure 1a, the fields marked in green indicate the possibility of PAS, while those marked in red indicate the possibility of antagonism.

Possible synergistic interactions were most often found after the combined action of the phages and imipenem (IMP), amoxicillin with clavulanic acid (AMC) and ampicillin (AMP), where an increase in the clear inhibition zones and in plaque size was observed in the case of most (eight) tested strains.

A decrease in the diameter of growth inhibition zones was observed after the combined use of norfloxacin (NOR) and the studied phages in eight *E. faecalis* strains.

The differences in the morphology of phage plaques in the antibiotic-active zone and in the antibiotic-free zone are highlighted in Figure 1b. The white arrows are used to indicate typical plaques of the phage strain, while the black arrows indicate plaques of increased size, obtained in the zone of the antibiotic action. The sample plates with differences in the size of the growth inhibition zones are presented in Figure 1c.

The results obtained enabled the selection of antibiotics (AMP, AMC, IMP) and *E. faecalis* strains (ATCC, C27, C13) for the subsequent tests.

### 2.3. Checkboard Assay

**Figure 2:**
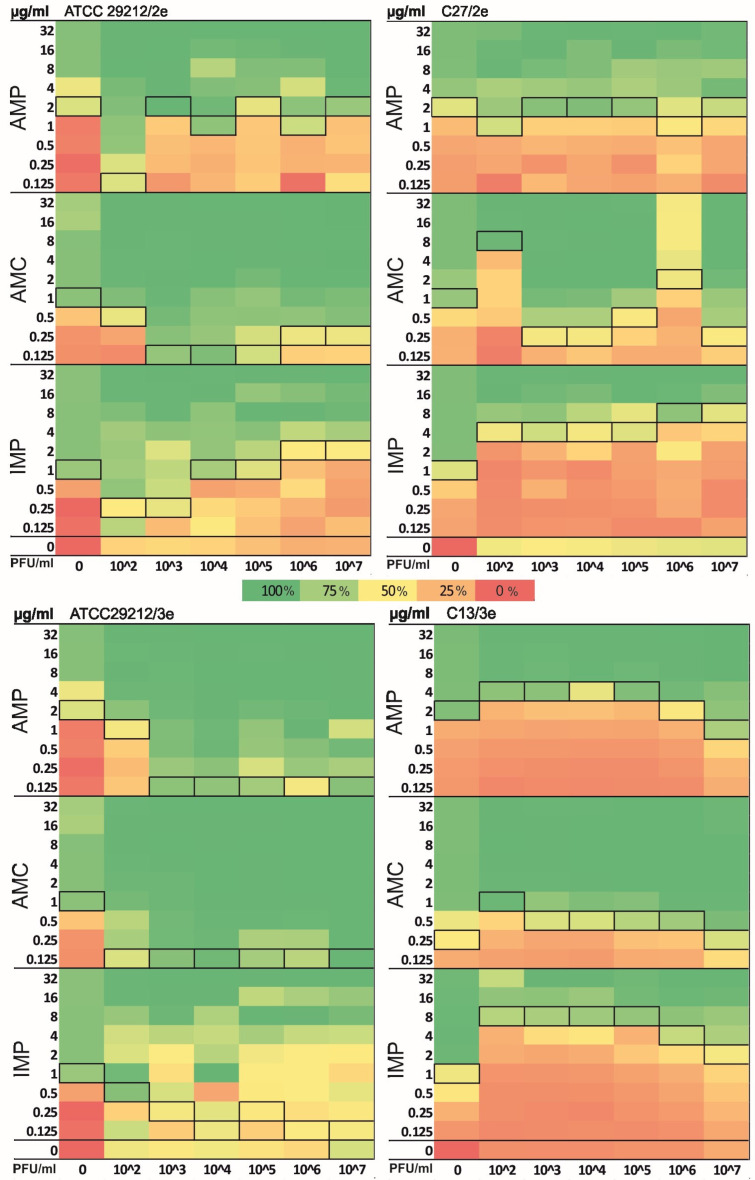
Heatmaps of checkerboard assay (after 24 h incubation) represent the mean *E. faecalis* strain growth inhibition percentage of each treatment. Fields marked with a black frame indicate MIC_50_ values. AMP—ampicillin, AMC—amoxicillin/clavulanic acid, IMP—imipenem.

Phages 2e and 3e with titers ranging from 10^2^ to 10^7^ plaque forming units per milliliter (PFU/mL), and antibiotics (AMP, AMC and IMP) at concentrations ranging from 32 to 0.125 µg/mL were tested. In the first stage of the study, the effect of each agent alone and then in combination was examined on the *E. faecalis* strains. Synograms were created (Figure 2), on which the reduction in bacterial growth is presented after the action of the tested factors (100% reduction means no bacterial growth and 0% reduction means growth equal to the positive control—bacterial culture without the addition of a growth-inhibiting factor). Additionally, MIC_50_ values are marked in the figure with black frames. Synograms made it possible to find points where the maximum bacterial killing effect was achieved and to determine the optimal antibiotic concentration and phage titers needed to inhibit the growth of *E. faecalis.*

The treatment with phage 2e alone was more effective at the titers of 10^2^–10^4^ PFU/mL than at the highest titer used—10^7^ PFU/mL. Phage 3e used in monotherapy at a sufficiently high titer (10^7^ PFU/mL) was able to reduce the growth of *E. faecalis* ATCC 29212 by an average of 61%. The phage was not effective against the C13 strain, where the reduction in growth was only about 2%.

The synograms of the studied strains showed a high reduction (more than 60%) when bacterial cells were treated with 2 µg/mL of AMP or 1 µg/mL of AMC and IMP.

The effect of the combined therapy can be seen in the middle section of the studied synograms. Numerous PAS phenomena between the tested agents were found, but synergistic interactions depended on the bacterial strain, phage and antibiotic which were applied. Both the studied phages, 2e and 3e, were found to interact synergically with all the antibiotics used. Such interactions were most frequently observed in the case of the *E. faecalis* ATCC 29212 strain with both phages 2e and 3e. The reduction in MIC_50_ values after phage 3e application was 16-, 8-, and 8-fold for AMP, AMC, and IMP, respectively. In the case of phage 2e, the reduction was 16-, 8-, and 4-fold for the same antibiotics. The results obtained suggest that the addition of phage reduced the effective MIC of all tested antibiotics. The lowest effective MIC values of the tested antibiotics were observed after the application of phage 2e at titers of 10^2^ and 10^3^ PFU/mL. In contrast, the best results for combined therapy with phage 3e were achieved with the titer of 10^7^ PFU/mL.

Antagonistic interactions between the tested preparations, mainly after the application of IMP and both phages 2e and 3e, were also detected. For example, an 8-fold increase in MIC_50_ values was observed after the application of phage 3e in the case of the *E. faecalis* C13 strain, and a 4- to 8-fold increase in MIC_50_ values was noted after phage 2e treatment against the *E. faecalis* C27 strain.

### 2.4. Time-Kill Kinetics

Additionally, the time–kill assay was performed for the system of AMP and the phages 2e or 3e to confirm the synergistic interactions observed in the checkerboard assay.

**Figure 3:**
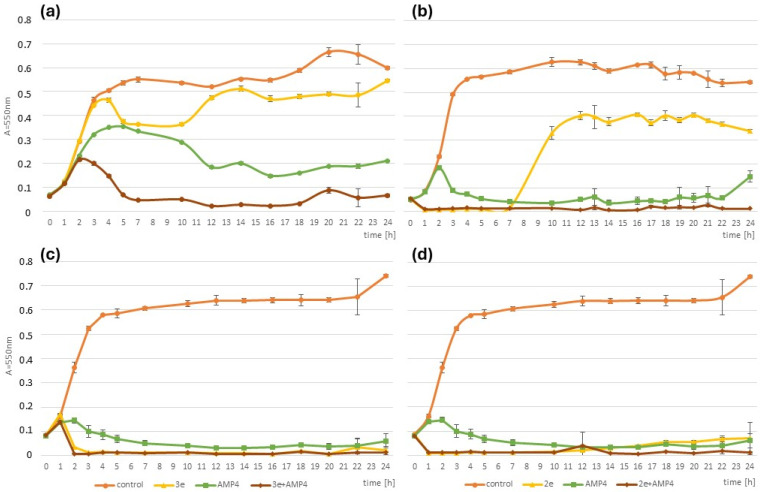
Time–kill assay of phage and antibiotic used individually and in combination against *E. faecalis* strains over 24 h. Phages vB_Efa29212_2e and vB_Efa29212_3e (multiplicity of infection, MOI 0.1), AMP (1/2 MIC), and their combination were used. *E. faecalis* strains: C13 (**a**), C27 (**b**), and ATCC (**c**,**d**) were tested. Statistical analysis: two-way ANOVA followed by Tukey’s post hoc test to identify statistically significant differences was performed.

The study demonstrated that phages and/or AMP effectively inhibited the growth of all tested strains. The synergistic effect of the combined treatment is shown in Figure 3a. The combination of agents was significantly more effective than monotherapy against the *E. faecalis* C13 strain (*p* ≤ 0.01) and showed a much stronger inhibitory effect (over 89.9% compared to the growth control) than phage or AMP used alone, which reduced the bacterial growth by 19.6% and 58.2%, respectively. Similarly, the combined therapy against the *E. faecalis* C27 strain was more efficient than using the phage alone (*p* ≤ 0.05) (Figure 3b). The strong growth inhibitory effect of the combined agents persisted throughout the experiment (for 24 h), whereas the inhibitory effect of the phage alone weakened over time (Figure 3a,b).

### 2.5. Frequency of Phage- and Antibiotic-Resistant Forms

**Figure 4:**
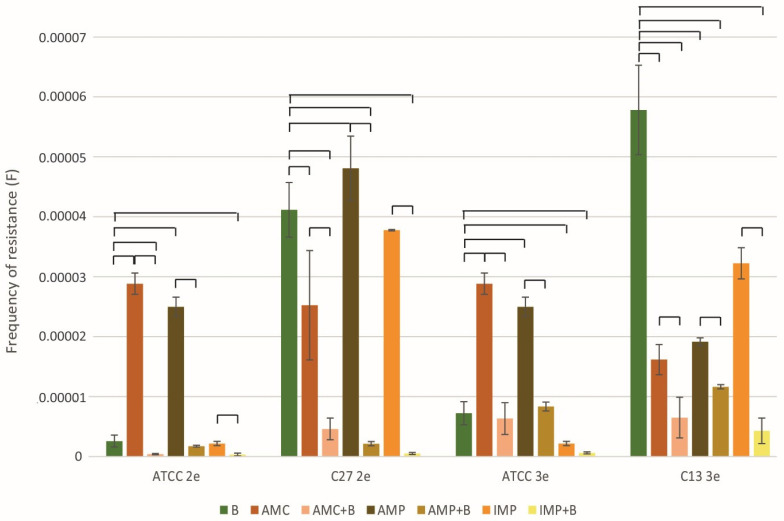
The number of spontaneous resistant variants of *E. faecalis* strains observed after treatment with phage and antibiotics. Phages vB_Efa29212_2e or vB_Efa29212_3e and antibiotics (AMP, AMC, IMP) were used individually and in combination. *E. faecalis* strains: C13, C27 and ATCC were tested. ANOVA II test, the Tukey post hoc test, was used for statistical analysis. Brackets indicate differences between mean values, with statistical significance defined as *p* ≤ 0.01.

The study compared the number of spontaneous resistant variants that emerged after the use of phages or antibiotics alone to those observed following the combined treatment (Figure 4). In the case of all tested strains, the combined use of antibiotics (AMC, AMP, and IMP) and phages (2e and 3e) resulted in a highly significant reduction in the number of resistant variants compared to those obtained after the use of either agent alone (*p* ≤ 0.01 or lower). For instance, the number of *E. faecalis* C13 variants resistant to 3e phage decreased after the introduction of IMP by approximately 41-fold. Similarly, the number of IMP resistant forms of *E. faecalis* C27 decreased by 76-fold after the introduction of phage 2e to the treatment.

The results obtained highlight the significant potential of combined therapy in limiting the development of *E. faecalis* resistance to both bacteriophages and antibiotics.

### 2.6. Characteristics of Phage Growth

**Figure 5:**
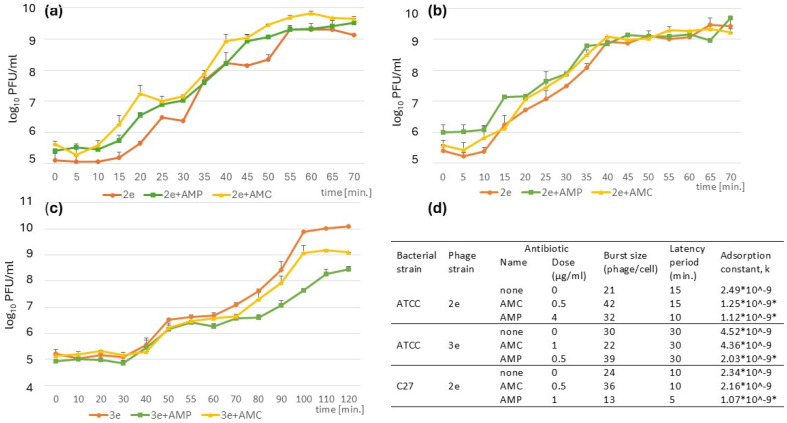
One-step growth curves of the phages vB_Efa29212_2e and vB_Efa29212_3e on a culture of *E. faecalis* strains: ATCC (**a**,**c**) and C27 (**b**), MOI 0.1 in BHI and BHI supplemented with 1/2MIC of AMP and AMC. (**d**) Biological properties of the phages (2e and 3e) propagated with or without sublethal doses of AMP or AMC: burst size, latency period, calculated on the basis of the one-step growth curves and the values of the adsorption constant, k. The nonparametric Mann–Whitney U test was used to compare the mean values of k with those obtained in controlled conditions (without an antibiotic), * *p* ≤ 0.05.

The one-step growth curves obtained after phages 2e and 3e cultivation under control conditions (in brain heart infusion (BHI) and in the medium supplemented with antibiotics: AMC and AMP at 1/2 MIC were compared and are presented in Figure 5a–c. The latent period and burst size of each phage under different cultivation conditions were determined (Figure 5d). For the 2e phage, the latent period ranged from 5 to 15 min and depended on the host bacterial strain and the presence of antibiotic supplementation. The addition of a subinhibitory concentration of AMP resulted in a 5 min reduction in the latency period—from 15 to 10 min when the phage replicated in *E. faecalis* ATCC 29212, and from 10 to 5 min when it replicated in the *E. faecalis* C27 strain. In contrast, the latency period of the 3e phage remained consistent; it was 30 min for all tested variants, which indicated no effect of antibiotic supplementation on that feature of the 3e phage. The burst size value ranged from 13 to 42 particles per bacterial cell for the 2e phage, and from 22 to 39 particles per bacterial cell for the 3e phage. For the phage 2e, AMC supplementation led to at least a 50% increase in the number of phages released from bacterial cells, regardless of the host strain.

Additionally, the adsorption constants (k) for the studied phages under different environmental conditions (with or without subinhibitory concentrations of AMC or AMP) were calculated and are presented in Figure 5d. The results demonstrated that the adsorption constants for both 2e and 3e phages decreased by approximately two-fold after AMP treatment (*p* ≤ 0.05). This reduction was not observed when a subinhibitory dose of AMC was used.

## 3. Discussion

*E. faecalis* bacteriophages are relatively understudied compared with phages that infect other pathogens, such as *Pseudomonas aeruginosa* or *Staphylococcus aureus* phages [3,21]. To effectively combat infections caused by multidrug-resistant *E. faecalis* strains, it is essential to characterize a greater number of phages. In this study, the potential of the two tested phages, vB_Efa29212_2e and vB_Efa29212_3e, for use in combination therapy with antibiotics against uropathogenic *E. faecalis* strains was demonstrated. The phages belonging to different families—2e to *Siphoviridae* and 3e to *Herelleviridae*—display diverse biological features and vary in their action against both planktonic and biofilm forms of *E. faecalis* [22,23]. The phages also have a different range in their hosts; while phage 3e replicates in many *E. faecalis* strains, phage 2e only in a few, but with high efficiency.

In the study, a higher efficacy of combined therapy compared to monotherapy against *E. faecalis* strains was observed. The phages were tested with seven antibiotics, and the best results were seen when they were paired with β-lactams, including ampicillin, amoxicillin/clavulanic acid, and imipenem. This suggests that the mechanisms of action of the phages and the antibiotics may enable synergistic interactions of these agents. The mode of action for β-lactam antibiotics is binding to inner membrane-bound enzymes known as penicillin-binding proteins (PBPs), which play a key role in bacterial peptidoglycan synthesis [24]. Additionally, these antibiotics block cell division, they disturb this process even at sublethal concentrations, which leads to the formation of elongated, filamentous cells that enlarge over time. Furthermore, β-lactams induce membrane instability (fragility) due to disruptions in the peptidoglycan structure [25]. These changes in the bacterial cell structure have an impact on the reproductive cycle of infecting bacteriophages, i.e., they influence their adsorption, reduce the lysis time, and increase the number of phages released from the cell [13,14]. The proposed mechanism for the increased phage adsorption rate following antibiotic treatment could involve a higher number of phage-targeting receptors on the bacterial cell wall. Consequently, the larger the bacterial surface area, the greater the number of receptors present [13,26]. Bullsico et al. [18] studied this phenomenon and created a mathematical model that demonstrated how sublethal doses of β-lactam antibiotics induced cell filamentation or swelling and promoted phage replication without altering the latency period. Additionally, the enlarged bacterial cells, with their increased cytoplasmic volume, provided more resources (a larger pool of precursors) for producing and assembling a greater number of virions. Changes in the peptidoglycan structure triggered by these antibiotics also made bacteria more vulnerable to phage lysis genes, such as lysozymes and holins [25]. In our study, we found that β-lactam antibiotics AMP and AMC influenced the reproductive cycle of the studied phages in different ways. After the application of sublethal doses of AMC, the results were similar to those obtained by Bullsico et al. [18]; the burst size of phage 2e increased, but the latency period remained the same as in the control conditions (environment without AMC). The use of sublethal doses of AMP had a different effect. A shortened latency period of phage 2e and a decrease in the adsorption constant of both phages were observed, and in two cases they were related to a higher number of released phages. The increased size of plaques in the antibiotic-affected zone also indicated the changes in the phages’ replication cycle, which were caused by the presence of antibiotics in the environment. It was documented that the plaque size is proportional to the adsorption rate and burst size and inversely proportional to the latent period [13,27,28]. Both antibiotics used, AMC and AMP, present a similar mechanism of action. They exhibit affinities to penicillin-binding proteins (PBPs), class A—transpeptidases (e.g., ampicillin has an affinity to PBP2s and PBP3s); it is possible that differences in the mode of action of these β-lactam antibiotics lead to distinct changes in bacterial morphology, because the extent of these alterations depends on the specific PBP targeted [29]. Thus, the selection of a specific β-lactam antibiotic affects the degree of filamentation or swelling, which in turn can influence the rate of phage adsorption by increasing the number of phage target receptors on the bacterial cell wall [13,30]. Moreover, phages belonging to different families respond differently to subinhibitory doses of antibiotics. Therefore, to use a phage–antibiotic combination in the therapy of bacterial infections, it is necessary to check each time how the tested agents interact and to select the phage dose and the starting concentration of the antibiotic to minimize antagonistic interactions and reduce the risk of failure in antibiotic–phage combination treatments.

The potential of phage–antibiotic combination therapy is broad, with demonstrated effectiveness against both planktonic bacteria and biofilms in a wide range of Gram-positive and Gram-negative species. Many authors reported high efficacy of phages and β-lactam antibiotic combinations. For instance, Papuashvili et al. [31] showed that using a β-lactam antibiotic, imipenem, along with a commercial bacteriophage preparation (*Pyobacteriophage*) significantly disrupted *Pseudomonas aeruginosa* biofilms. Lu et al. [32] also observed the best synergy when combining phages with β-lactam antibiotics against the planktonic and sessile form of *Shigella* strains. Bedi et al. [33] also showed that a combination of amoxicillin and phage caused a significant reduction in *Klebsiella pneumoniae* biofilm and suggested that the phages could be used successfully along with antibiotic therapy. Unfortunately, there are also reports of antagonistic effects of β-lactams and phages, e.g., Ma et al. [34] discovered the antagonistic effect of phages and AMCs on *Salmonella* in piglet and mouse models. A decreased bacterial susceptibility to AMC and inhibition in the phage plaque formation after combined treatment were noted. There is still limited research on the mechanism of antagonism between β-lactam antibiotics and phages. It is known that the development of bacterial resistance to phages leads to changes in bacterial physiology or an increase in their virulence, which can result in cross-resistance to antibiotics. For instance, bacteria resistant to phages can produce β-lactamases more efficiently, leading to increased resistance to β-lactam antibiotics [16]. While many studies focus on *P. aeruginosa*, *E. coli* and *S. aureus* [30,31,35], research on the use of combined therapy especially with β-lactam antibiotics against *Enterococci* is less common; therefore, the results of this work are expected to help supplement the knowledge in this field.

We proved that the tested phages reduced the dose of antibiotics needed to inhibit the growth of *E. faecalis*, and the use of combined therapy led to a significant reduction in the number of forms resistant to the agents used.

In this study, the checkerboard assay made it possible to determine the synergistic interactions between phage 2e or 3e and AMP and AMC which led to a significant reduction in the MIC_50_ values. Likewise, the combination of phages UP17 or Jk08 or 113 and meropenem resulted in a decrease in the antibiotic MIC by more than two-fold for 24, 34 or 26 of 100 *E. coli* strains [36]. A similar effect was observed after a treatment with phage vB_Sau_S90 and four antibiotics against methicillin-resistant *S. aureus*. The combination of the phages and antibiotics (fosfomycin, ciprofloxacin, oxacillin or vancomycin) reduced the MIC by multiple folds compared to individual treatment with phages or antibiotics. The MIC reduction was the greatest for oxacillin and ranged from 8 μg/mL to 0.25 μg/mL [37]. In the study conducted on the antibiotic-resistant *E. coli* O157 strain, Moradpour et al. proved that the synergistic action of phage (gT0E.co-MGY2) and ampicillin was the most potent and caused a 95% growth inhibition of bacteria [35]. What is more, a combination treatment led to the resensitization of bacteria to antibiotics, e.g., AMP and AMC. The better effect of combinatorial compared to individual treatments (with phages or antibiotic) was confirmed by a time–kill analysis. Importantly, the effectiveness of the combination was maintained for up to 24 h while treatment with phages alone often declined over time, e.g., due to bacteria developing resistance to phages [38].

What is worth mentioning, in the case of the phage 2e and β-lactams action against the studied *E. faecalis* strains, synergistic interactions occurred when the phage preparation was administered at a low titer. In contrast, there was no such tendency in the case of phage 3e, where a greater reduction in the MIC_50_ values was obtained after the use of high titers of the phage. Studies conducted on *E. coli* phages showed that the best killing effect was caused by those PAS systems that consisted of phages with a titer of 10^5^–10^9^ [39]. On the other hand, the studies by Gordillo Altamirano et al. [40] on the effect of phages and antibiotics on *Acinetobacter baumannii* strains showed the strongest PAS effect when using phages with titers of 10^6^–10^7^. The results of these studies show the importance of appropriate phage titer administration when using combined PAS therapy. Higher virus titers are not always the most effective in therapy, and could even result in antagonistic interactions.

The highly malleable genome of *E. faecalis* allows the bacteria to quickly adapt to changing environmental conditions [7,8]. Thus *E. faecalis* easily develops phage resistance, usually as a result of mutations in polysaccharides or membrane protein genes associated with the cell wall [3,41]. The high mutation rate emphasizes the need to carefully select the right therapy to prevent the development of resistant strains. PAS could be a promising alternative to effectively control *E. faecalis* resistance to commonly used antibiotics. In our study, we demonstrated that for all tested strains, the combined treatment of β-lactam antibiotics (AMC, AMP, and IMP) and the studied phages significantly reduced the number of variants resistant to both phages and antibiotics. Similar results can be found in the available literature. Valerio et al. [42] demonstrated that the combined action of phages and antibiotics (e.g., ampicillin) resulted in the same or lower resistance in *E. coli* strains compared to the use of each treatment separately. Likewise, Jo et al. [43] confirmed in their studies that the simultaneous use of bacteriophages and antibiotics generated *S. aureus*-resistant forms less frequently than when used individually. Bulssico et al. [18] also observed that introducing even a small number of bacteriophages significantly reduced the mutagenesis rate across the entire bacterial population. The literature data confirm that PAS reduces resistance to both antibiotics and phages, lowering the risk of resistance [40]. Researchers frequently link this phenomenon to a mechanism known as “evolutionary trade-off”. This mechanism suggests that when bacteria are under phage selection pressure, they may mutate in a way that results in their resensitization to antibiotics [12].

Phage therapy has significant potential but requires further investigation before it can be implemented in clinical practice. An expert panel [44], considering its clinical application in patients, highlighted the need for comprehensive research on various aspects, including dosage, administration methods, therapy duration, and interactions with antibiotics and the host immune system. The experts focused on the importance of both individualized phage therapy and synergistic phage–antibiotic therapy, emphasizing the necessity to develop standardized and precise methodologies for its effective study.

Our in vitro study highlights the potential of phage–antibiotic combination therapy to combat *E. faecalis* infections. The synergistic effects between β-lactam antibiotics and specific phages enabled the use of lower antibiotic doses after phage application to inhibit *E. faecalis* growth and a significantly reduced the emergence of resistant variants of the bacteria. Phage 2e demonstrated synergy with β-lactams at low titers, while phage 3e required higher titers to achieve similar reductions in the MIC_50_ values. Furthermore, despite similar mechanisms of action, different antibiotics may have different effects on phage replication. These findings confirm the importance of the careful selection of both phages and antibiotics, as well as their appropriate dosages, in combination therapies to maximize efficacy and minimize resistance development. Future studies should focus on the mechanism of interaction between phages and β-lactam antibiotics to support the implementation of this therapy.

## 4. Materials and Methods

### 4.1. Bacteria and Bacteriophage Strains

Phages vB_Efa29212_2e and vB_Efa29212_3e were isolated from municipal sewage from the Group Wastewater Treatment Plant in Lodz, Poland and stored at 5 °C. For experiments, phages were propagated in Brain Heart Infusion (BHI, BTL, Lodz, Poland) on the host *E. faecalis* ATCC 29212. For this purpose, the bacteria were diluted to 10^7^ CFU/mL and phages were added so that the number of virions was 10^6^ PFU/mL (multiplicity of infection—MOI 0.1). The suspension was incubated for 3 h at 37 °C, 150 rpm/min and through the night at 5 °C. Then, the suspension was filtered using 0.2 µm membrane filters (Sartorius, Göttingen, Germany). The double agar overlay plaque assay was used to count the bacteriophages. The studied bacteriophages had been previously characterized [22].

Bacterial strains of *E. faecalis* used in the study included the reference ATCC 29212 and strains isolated from urological catheters of long catheterized patients which were kindly donated by the outpatient clinic of M. Pirogow Specialist Hospital in Lodz, Poland. The patients were men over 70 years old suffering from prostatic hyperplasia. In cases of UTIs, patients were treated mainly with ciprofloxacin.

Bacteria were stored at −80 °C in L-Broth (BTL, Lodz, Poland) supplemented with 10% DMSO (Avator, Gliwice, Poland). During the experiments the strains were cultivated on Brain Heart Infusion Agar (BHA, BTL, Lodz, Poland) plates and next a single colony was inoculated to 6 mL of BHI and incubated for 20 h at 37 °C.

### 4.2. Determination of the Bacterial Host Profile

The host range of the studied bacteriophages was determined using the spot method. A total of 33 *E. faecalis* strains were tested. A measurement of 10 µL of bacteriophage lysates (titer 2 × 10^9^ PFU/mL) were dropped on the double-layer soft agar premixed with 100 µL of a bacterial strain. The plates were incubated for 20 h at 37 °C and a clear zone at the site of application indicated that the bacterial strain was a host for the phage. The reading was carried out using a 4-point scale, where 4 represents a fully cleared zone, 3 indicates an almost fully cleared zone, 2 denotes a partially cleared zone with low turbidity, 1 signifies a partially cleared zone with high turbidity, and 0 corresponds to a zone with turbidity identical to the bacterial lawn.

Additionally, an RTD test was performed to determine the maximum dilution of the phage lysate, whose application caused complete clearing zones of the host bacterial strain. For this purpose, 20 µL of successive dilutions of the phage lysate (ranging from 1:10 to 1:10,000,000) and BHI (BTL, Lodz, Poland) as a negative control were placed on a double-layer soft agar premixed with a bacterial strain. The plates were incubated for 24 h at 37 °C and RTD was determined.

### 4.3. Modified Disk-Diffusion Method for Antibiotic–Phage Synergy Testing

A modified double-layer agar disc diffusion test method was used to study the presence of phage–antibiotic synergy [45,46]. Samples of 200 µL of phage lysate and 100 µL of bacteria (OD = 0.5) were added to 3.2 mL of molten agar cooled to 45 °C (BTL, Lodz, Poland) and poured onto a plate. The phage titer was selected empirically to ensure an appropriate number of plaques on the double agar plate, aiming for a balance where single plaques were clearly visible, avoiding both an excess and a deficiency of plaques. Then, antibiotic discs were placed on the plate. The antibiotic discs (Biomaxima, Lublin, Poland) with standard concentrations specified by the European Committee on Antimicrobial Susceptibility Testing (EUCAST) guidelines were used: ampicillin (AM 30), amoxicillin with clavulanic acid (AMC 30), ciprofloxacin (CIP 5), vancomycin (VA 5), norfloxacin (NOR 10), teicoplanin (TEC 30), imipenem (IMP 10). Additionally, proper control double-agar plates only with phage (without antibiotics) and plates with antibiotics alone (without the addition of phage) were prepared. The plates were incubated for 20 h at 37 °C. Then the zones of bacterial growth inhibition around the antibiotic discs were measured with a digital caliper. Changes in plaque size near the sub-inhibitory concentration zone were observed.

### 4.4. Minimum Inhibitory Concentration (MIC) Determination

MICs for the antibiotics were determined using the EUCAST reference method—the broth microdilution method with some modifications [47,48]. The experiment was performed in BHI medium (BTL, Lodz, Poland) to standardize the conditions of all experiments in this study. The studied phages of *E. faecalis* were stored and propagated in BHI medium. The experiments were performed in triplicate for selected *E. faecalis* strains and the reference *E. faecalis* ATCC 29212 strain was used as a control.

### 4.5. Checkerboard Assay for Antibiotic–Phage Synergy Testing

The assay involved mixing phages and antibiotics at many titers and concentrations. For the experiment, *E. faecalis* was cultivated in BHI (BTL, Lodz, Poland) for 20 h at 37 °C. The bacteria were then diluted in BHI to achieve a concentration of 1 × 10^7^ CFU/mL. Antibiotics (AMC, AMP, and IMP, Merck Life Science, Darmstadt, Germany) at ½ MIC in BHI and phages with titers from 1 × 10^7^ to 1 × 10^2^ in BHI were prepared. A series of two-fold antibiotic dilutions in BHI containing phages at the appropriate titers were made in a 96-well plate (Anicrin, Scorzè (VE), Italy). Subsequently, 100 µL of bacterial suspension was added to the wells, to obtain the MOI from 1 to 0.00001 [49]. Additionally, controls of the sterility of antibiotics and phages were prepared, as well as controls of bacterial growth in BHI (without killing agents). The wells, where the effects of single agents (phage or antibiotic) on bacterial growth were tested, were also included in the test. The plates were incubated in a humid chamber for 20 h at 37 °C. The growth of microorganisms was monitored by measuring absorbance at 550 nm using a Multiskan GO reader (Thermo Fisher Scientific, Vantaa, Finland). The percentage of bacterial growth inhibition by the combined or individual action of the agents was calculated, with 100% corresponding to the bacterial growth control.

Growth inhibition [%] = 100 − (100% ∗ OD_treatment_/OD_growth control_)

MIC_50_ values were determined, i.e., the minimum concentration of antibiotic inhibiting the growth of 50% microorganisms.

### 4.6. Time–Kill Assay

The study evaluated the activity of antimicrobial agent, ampicillin (Merck Life Science, Darmstadt, Germany), phages 2e or 3e, as well as the combination of phages with ampicillin against the *E. faecalis* strains over a 24 h period. For the experiment, 20 h cultures of *E. faecalis* strains were diluted in BHI (BTL, Lodz, Poland) to achieve a concentration of 2 × 10^7^ CFU/mL. Phage lysate was diluted in BHI to reach a density of 2 × 10^6^ PFU/mL, ensuring an MOI of 0.1 when mixed with the bacterial culture. Ampicillin was prepared at a final concentration of ½ MIC in BHI. The bacterial culture and antibacterial agents were mixed together in equal volumes on a type F polystyrene plate (Anicrin, Scorzè (VE), Italy), with a final volume of 200 μL. The plate was incubated for 24 h at 37 °C. The absorbance was measured at one-hour intervals on a plate reader (Multiskan, Go, Thermo Fisher Scientific, Vantaa, Finland), at a wavelength of λ = 550 nm. Measurements began as soon as all the tested systems were introduced to the plate. The positive control was a bacterial culture in BHI medium, while sterile BHI medium served as a negative control.

### 4.7. Frequency of Phage- and Antibiotic-Resistant Forms (Spontaneous Mutants)

The frequency of resistance of *E. faecalis* strains to the studied phages and antibiotics (AMP, AMC, IMP, Merck Life Science, Darmstadt, Germany) was examined. Bacterial suspensions of 1 × 10^8^ CFU/mL in BHI, a phage lysate with a titer of 1 × 10^8^ PFU/mL in BHI (BTL, Lodz, Poland) and the tested antibiotic at a concentration of 2 MIC in BHI were prepared for the experiment. The double agar overlay plaque assay was used. For this purpose, 100 µL each of bacteria, phage and/or antibiotic and/or BHI was added to 3.2 mL of dissolved agar, so that the final volume was 3.5 mL. The agars were poured onto BHA (BTL, Lodz, Poland) plates and then incubated for 48 h at 37 °C. The resistant forms of the grown bacteria were counted. Bacteriophage-resistant forms were determined on plates where the bacteria, phage and BHI were mixed. Antibiotic-resistant forms were tested on the plates with bacteria, an antibiotic and BHI and bacteriophage-antibiotic-resistant forms were examined on the plates with bacteria phage and an antibiotic. The frequency of resistance (F) to each phage and antibiotic was calculated according to the following formula:

F = n/N,

where n—average number of resistant colonies; N—initial bacterial culture density CFU/mL [43,50,51].

### 4.8. One-Step Growth Curve and Phage Adsorption Rate

One-step growth curves and adsorption rates of the studied phages were determined after the incubation of the phage with the host bacterial strain under optimal conditions in BHI (BTL, Lodz, Poland) and in BHI with the addition of antibiotics (AMC and AMP, Merck Life Science, Darmstadt, Germany) at a concentration of ½ MIC. The experiments were conducted as previously described with some modifications [51,52,53]. Briefly, 1 mL of a 20 h culture of the appropriate *E. faecalis* strain in BHI, with a fixed density, was centrifuged at 13,000× *g* for 5 min at room temperature (Sigma 1-15, Osterode am Harz, Germany). The obtained cell pellet was washed in SM buffer (50 mM Tris-HCl (Serva, Heidelberg, Germany), 100 mM NaCl (Eurochem, Tarnow, Poland), 0.01% gelatin (Sigma, St Louis, MO, USA), and 8 mM MgSO_4_ 7H_2_O (Chempur, Piekary Slaskie, Poland) and then resuspended in 5 mL of SM or SM with the addition of an appropriate antibiotic (at a concentration of ½ MIC) to achieve a bacterial culture density of 2 × 10^8^ CFU/mL. The number of bacterial cells added in the experiment was additionally verified, the prepared suspension was diluted and spread onto BHA (BTL, Lodz, Poland) plates (CFU/mL).

Phages were diluted in SM buffer to obtain a titer of 2 × 10^6^ PFU/mL so that the bacteria and phages could be mixed at MOI of 0.01. Immediately after mixing, the bacteriophages were counted by the double agar overlay plaque assay to determine the initial number of phage particles. The suspension was incubated for 5 min at 37 °C in a water bath. Then it was centrifuged at 13,000× *g* for 5 min (Sigma 1-15, Osterode am Harz Germany). The obtained supernatant was filtered through a 0.22 µm syringe filter (Sartorius, Göttingen, Germany) and the unabsorbed bacteriophages were counted, using the double-agar method. The obtained pellet was washed in SM buffer, and then resuspended in 20 mL of BHI broth (or BHI with an antibiotic at a concentration of ½ MIC). The suspension was incubated for 70 min or 120 min for 2e and 3e, respectively. Samples were taken at 0 h and at 5 min or 10 min intervals for 2e and 3e, respectively, and the double agar overlay plaque assay was performed using BHA plates. The plates were incubated for 24 h at 37 °C and the phage titers were calculated. The latency period, burst size and adsorption rate were then determined using the following formulas:

Burst size: PFU/infected cell = max phage yield/initial phage yield (t0) [54].

The adsorption constant is as follows:

k = (−1/B × t) × ln (P/P0),

where k is the adsorption rate constant (mL/min); P is the concentration of free phages per mL; P0 is the initial phage concentration; B is the initial bacterial concentration; t is the time (min) [55,56].

### 4.9. Statistical Analysis

Means and standard deviations (SD) were calculated from a minimum of three independent repetitions of each experiment. Statistical analysis involved several tests: the Shapiro–Wilk test to assess normality, Levene’s test to evaluate the homogeneity of variance, the two-way ANOVA test (ANOVA II) and the Tukey post hoc test to identify statistically significant differences between more than two independent groups. Additionally, the nonparametric Mann–Whitney U test was employed to compare differences between two independent groups for data that were not normally distributed. Differences between the means of the two groups were deemed significant at *p*-values ≤ 0.05, ≤0.01 and ≤0.001. Statistical analysis was performed using Statistica 13.3 software (StatSoft Inc., Kraków, Poland).

## Figures and Tables

**Table 1:** The *E. faecalis* host range of vB_Efa29212_2e and vB_Efa29212_3e bacteriophages.

Bacterial Strain	vB_Efa29212_2e		vB_Efa29212_3e	
	Spot Test	RTD	Spot Test	RTD
ATCC29211	4	10^−3^	4	10^−3^
C3_1	0	0	0	0
C3_3	0	0	2	10^0^
C7	0	0	2	10^0^
C8	0	0	4	10^−2^
C9	0	0	2	10^0^
C12	0	0	1	10^0^
C13	0	0	4	10^−3^
C15	0	0	1	10^0^
C16	0	0	4	10^−2^
C20	0	0	3	10^−2^
C21	0	0	3	10^−2^
C23	0	0	2	10^0^
C27	4	10^−3^	4	10^−1^
C30	0	0	3	10^−1^
C31	0	0	1	10^0^
C37	0	0	1	10^0^
C44	0	0	2	10^0^
C46	0	0	1	10^0^
C51	0	0	1	10^0^
C58	0	0	1	10^0^
C61	0	0	4	10^−2^
C63	1	10^0^	0	0
C67	0	0	4	10^−2^
C69	0	0	1	10^0^
C71	0	0	1	10^0^
C73_1	2	10^0^	3	10^−1^
C73_2	0	0	4	10^−2^
C80	0	0	1	10^0^
C83	0	0	3	10^−1^
C84	0	0	3	10^−2^
C85	0	0	1	10^0^
11.3	0	0	3	10^−1^

0, 1, 2, 3, 4—degree of lysis (where 4 indicates a complete clearing zone, 3—an almost complete clearing zone, 2—an incomplete clearing zone with low turbidity, 1—an incomplete clearing zone with high turbidity, and 0—a zone with turbidity identical to the bacterial lawn) RTD—routine test dilution (the highest phage lysate dilution at which complete clearing zones of bacteria strains was observed).

## Data Availability

Data are contained within the article.
